# Artificial intelligence in action: building simulation and analysis tools for powder diffraction

**DOI:** 10.1107/S2053273325007508

**Published:** 2025-09-04

**Authors:** Paolo Scardi, Marcelo A. Malagutti

**Affiliations:** a Department of Civil, Environmental and Mechanical Engineering University of Trento via Mesiano 77 Trento 38123 Italy; Institute of Crystallography - CNR, Bari, Italy

**Keywords:** X-ray diffraction, artificial intelligence, LLMs, large language models, machine learning, software development

## Abstract

This paper explores the application of generative pre-trained transformer (GPT)-based large language models (LLMs) in the development of simulation and analysis tools for X-ray powder diffraction. We demonstrate how these models enable users with minimal programming experience to generate functional and efficient code through natural language prompts. The discussion highlights both the capabilities and limitations of LLM-assisted coding, offering insights into the practical integration of artificial intelligence for simulating and analysing simple X-ray powder diffraction patterns.

## Abstract 2

The present work demonstrates the use of large language models for generating computational tools to aid X-ray powder diffraction analysis.

Artificial intelligence (AI), including machine learning (ML) and deep learning (DL), is transforming powder diffraction by enhancing data interpretation and automating complex tasks. For instance, Yanxon *et al.* (2023 ▸) employed gradient boosting methods to effectively identify single-crystal diffraction spots from X-ray diffraction images, reducing manual intervention and processing time. Likewise, Ozaki *et al.* (2025 ▸) and Guo *et al.* (2025 ▸) developed convolutional neural networks (CNNs) capable of predicting space groups and unit-cell volumes from powder X-ray diffraction profiles, highlighting the potential of DL in structural characterization. Schuetzke *et al.* (2021 ▸) trained CNNs on synthetic diffraction patterns to identify phases in complex mixtures, achieving high accuracy and robustness against variations in experimental conditions. These are some of the applications that streamline the analysis of large datasets which is particularly beneficial in high-throughput experiments.

Beyond data analysis, AI is increasingly contributing to the development of protocols and software tools that support powder diffraction studies. The *XRDplayground* (Estrada & Malfatti-Gasperini, 2025 ▸) open-source Python-based educational tool exemplifies this trend. It enables users to simulate unit cells and their corresponding powder X-ray diffraction patterns interactively, illustrating AI’s expanding role in both research and education within the field. In the present work, we explore the use of *ChatGPT-4o*, a generative pre-trained transformer (GPT) large language model (LLM) (OpenAI, 2025 ▸), to assist in the development of software for the simulation and potential analysis of powder diffraction patterns. This application exemplifies a growing trend in which AI not only supports data interpretation but also contributes directly to the creation of computational tools. While the process was not without challenges – such as the need for precise prompting, iterative debugging and careful validation – the overall experience proved effective and time-efficient. *ChatGPT* enabled rapid prototyping across multiple programming environments, facilitating smooth transitions between Python, C++ and CUDA. These results underscore the potential of AI-driven code generation as a flexible and powerful approach to accelerating software development in crystallography.

This study aimed not to produce finalized software, but to assess the potential and limitations of *ChatGPT* as a programming assistant for scientific coding. As a representative test case drawn from our area of expertise, we focused on the modelling and simulation of powder diffraction data based on atomistic models. Effective interaction with the AI required carefully formulated, detailed prompts and was most productive when conducted through iterative exchanges – gradually refining intermediate outputs and addressing emerging inaccuracies. To ensure computational autonomy, all generated codes were designed for offline execution on local systems. Given the model’s tendency to favour generality and brevity, the resulting content, particularly equations and syntax, was subject to manual verification and correction, often through further prompting. This methodology illustrates both the practical utility and current constraints of *ChatGPT* as a co-developer in the context of scientific computing.

A poorly structured question, asking to ‘produce a plot with the simulation of a X-ray powder diffraction pattern from a powder made of identical spheres of Pd, about 6 nm diameter’ yields a disappointing answer: a schematic powder pattern, with a few peaks of normalized intensity and width resulting from the Scherrer equation. The result improves significantly if we ask to use an atomistic model of spherical nanocrystals, with a Debye–Waller (DW) factor for thermal effects and the atomic scattering factor (*f*). For the latter it was necessary to specify the Cromer–Mann coefficients for Pd, to use the same ones as *TOPAS* (Coelho, 2018 ▸), the reference software used to verify all the generated scripts. The Python code is simple and efficient: in 114 lines it includes the generation of the atomic coordinates inside a sphere, calculates the distances of all pairs and calculates the Debye scattering equation (DSE). The code and all the prompts used are available in Section 1 of the supporting information (SI) file.

Next, we asked in a second *ChatGPT* prompt to write a script that treats the simulated pattern as unknown data, identifies peaks, fits them using the Pawley method, and refines both lattice parameter and domain size. Based on the result, we asked to identify the material. In this analysis, which requires indexing the pattern, it was suggested that the structure was cubic, and to use pseudo-Voigt (pV) line profile functions and a Chebyshev polynomial of degree 3 or 4 for the background. The code and prompt are available in Section 2 of the SI.

The result [Fig. 1 ▸(*a*)] is good, considering the simplicity of the script and the fact that Voigtian profile functions cannot model in detail the peaks of a DSE-generated pattern for an atomistic crystallite model (Beyerlein *et al.*, 2011 ▸). The unit-cell parameter given by the peak positions (*a* = 3.8913 Å) and the crystallite size (53.8 Å) obtained with the Scherrer equation from the pV peak width are close to the values set in the simulation. *ChatGPT* correctly identified the material as palladium.

To improve the quality of the fit, *ChatGPT* was asked to replace the Voigtian profile with that for a spherical crystallite of diameter *D* (Langford & Wilson, 1978 ▸; Patterson, 1939 ▸), to be refined by least squares:
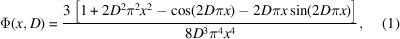
with 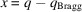, where 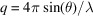 is the modulus of the scattering vector and 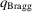 its value in the Bragg condition. As shown in Fig. 1 ▸(*b*), the modelling improves significantly (*R*_wp_ goes from 3.62% to 1.28%) and refines a diameter value (*D* = 59.98 Å) almost identical to the simulated one. The code used for the fitting is given in Section 3 of the SI. The agreement with the input data can still be improved by prompting additional details to the modelling (*e.g.* small-angle X-ray scattering, which is included in the simulation with the DSE), but it does not change the concept we wanted to show, which is that AI can support fast and effective programming of typical powder diffraction tools.

For a more realistic model, we asked *ChatGPT* to simulate (i) thermal disorder via random atomic displacements and (ii) microstrain via lattice parameter variation among spherical crystallites. To test this (Section 4, SI), we used 10 random displacements and 30 bins to model a 1% microstrain distribution in 50 Å spheres. Rietveld refinement [Fig. 2 ▸(*a*)] used whole powder pattern modelling (WPPM) macros in *TOPAS* (Scardi *et al.*, 2018 ▸; Coelho, 2018 ▸), returning the simulated 5 nm diameter and 1% microstrain as result, similarly to small-box pair distribution function (PDF) refinements [Fig. 2 ▸(*b*)] (see details in Section 5, SI). However, *ChatGPT* mistakenly used 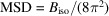 (MSD is mean-square displacement) per Cartesian direction during the temperature emulation, requiring manual correction from the user, dividing the DW value by three.

DSE calculations are computationally intensive (scaling with the square of the number of atoms), but optimizations like binning, a known strategy since the 1940s (Germer & White, 1941 ▸) and present in the *Debyer* software (Wojdyr, 2020 ▸), and GPU acceleration, like in *Powdog* (Gelisio *et al.*, 2010 ▸), can speed its calculation. With this in mind, we benchmarked another five simulation strategies to compare *ChatGPT* capabilities with conventional simulation software: (1) brute-force DSE for one 5 nm face-centred cubic (f.c.c.) Pd sphere, (2) pair distance binning with thermal modelling, (3) parallelization of the microstrain calculation associating one microstrain step for each thread, (4) simulating a log-normal distribution of diameters with parallelization, and (5) combining strategies (2), (3), (4) using CUDA. With the patterns simulated, structural and microstructural refinements were performed using Rietveld and WPPM modelling in *TOPAS*, with values reported in Table 1 ▸. The profile modelling and prompts are given in Sections 6–9 of the SI. Python required memory fixes, C++ needed manual corrections to the parallelization procedure, while CUDA had issues in CPU-to-GPU data allocation and vice versa. Nevertheless, GPUs provided ∼10× speedup for single-sphere simulations, with each thread computing one 2θ point [comparing approach (1) in Table 1 ▸]. Despite CUDA’s efficiency [fivefold faster than *Powdog* comparing approach (1) in Table 1 ▸] *Debyer* still surpasses *ChatGPT*’s binning strategy [see approach (2) in Table 1 ▸], showing that while LLMs offer adaptable coding, expert-developed software remains more efficient.

In Table 1 ▸, we present a stepwise increase in model complexity across the study cases. This incremental refinement is essential when generating complex algorithms via LLMs, as it helps mitigate their limited accuracy and ensures more reliable performance. Indeed, the accuracy of LLMs is doubling yearly, with performance scaling as a power law with model size, dataset and training compute (Kaplan *et al.*, 2020 ▸). However, *ChatGPT* still lacks built-in mathematical validation for equations and unit consistency, unless explicitly prompted, generating imprecise physical models for the simulations, requiring each step of the simulation to be manually verified. The LLM model is trained on petabytes of internet data using billion-parameter architectures to predict the most statistically likely output based on its database patterns (OpenAI, 2025 ▸). For complex scientific tasks, such as simulating X-ray diffraction of palladium nanospheres, accuracy depends on the presence of similar examples in its training set, generating less precision for less investigated scientific fields. One strategy used to mitigate this is to compare responses from multiple LLMs or to use real-time web searches, though this introduces risks if online sources are physically incorrect. For instance, the early Python code from *ChatGPT* systematically misapplied the DW factor due to an incorrect equation sourced from Wikipedia (Wikipedia, 2024 ▸). Nonetheless, when prompted precisely, *ChatGPT* can accurately cite sources and reproduce established equations and algorithms. Understanding and improving the models for structural and microstructural characterization is still a challenge in X-ray diffraction and PDF analysis, with numerous works recently devoted to that topic (Dinnebier & Scardi, 2023 ▸, 2021 ▸; Terban & Billinge, 2022 ▸; Scardi & Malagutti, 2024 ▸; Malagutti *et al.*, 2025 ▸). As a coding assistant, LLMs significantly accelerate scientific software development, even for users without extensive programming experience. However, careful human oversight remains essential to ensure correctness in algorithm implementation and physical modelling.

## Supplementary Material

Supporting information file containing all the prompts and codes used in the main text. DOI: 10.1107/S2053273325007508/ae5165sup1.pdf

## Figures and Tables

**Figure 1:**
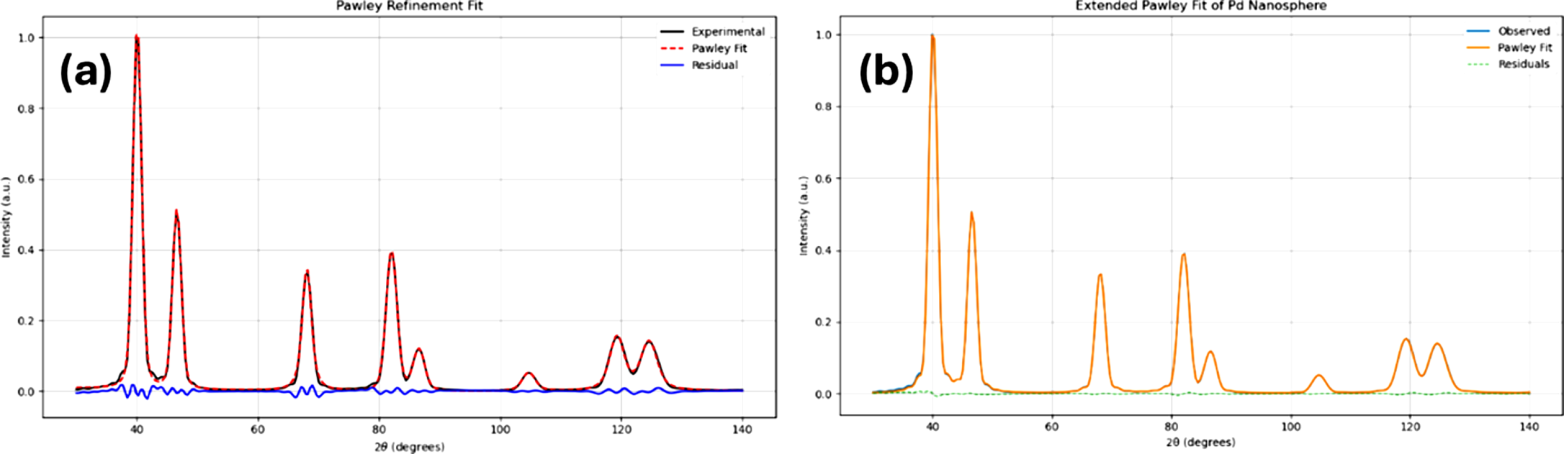
(*a*) Pawley method with pV line profiles: *a* = 3.8913 Å, crystallite size (from peak 111) = 53.8 Å, *R*_wp_ = 3.62%; (*b*) same using equation (1) for the line profile: *a* = 3.8914 Å, crystallite diameter = 59.98 Å, *R*_wp_ = 1.28%. Input data generated from atomistic simulation for 60 Å spheres of Pd (see text for details).

**Figure 2:**
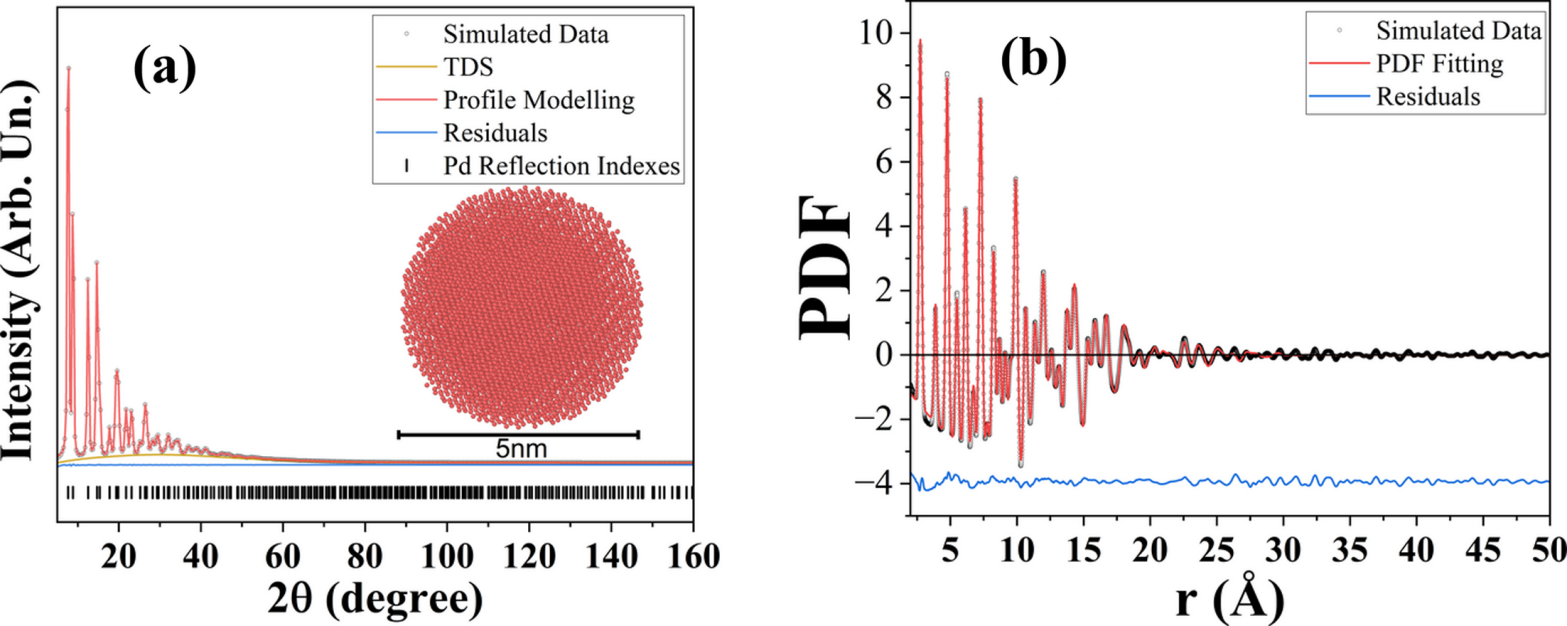
*TOPAS* analysis: (*a*) Rietveld refinement and (*b*) PDF analysis. The open black dots correspond to the simulated data, red lines to the profile modelling/fitting, blue lines to the residual. The thermal diffuse scattering (TDS) contribution is given by the yellow line, and the thick black markers correspond to the indexed reflections of Pd.

**Table 1:** List of simulations and profile modelling results using WPPM analysis

Simulation	Language	Execution time (min)	Strategy	Crystallite diameter (nm)	Microstrain ×10^2^	DW coefficient (Å^2^)
(1)	Python	18.4	(no binning)	5.03653	–	–
	C++	22	(no binning)	5.0038	–	–
	CUDA	0.1530	(no binning) + GPU	4.9945	–	–
	*Powdog*	1.3	No binning + GPU	4.9962	–	–
(2)	Python	4.59	Binning	5.02757	–	0.2927
	C++	3.78	Binning	5.002	–	0.2991
	CUDA	0.31	(no binning)	4.988	–	0.2969
	*Debyer*	∼5 s†	Binning	4.9985	–	–
(3)	Python	8.13	Binning + parallelization	5.02143	0.21730‡	0.2949
	C++	5.99	Binning + parallelization	5.01548	0.2156‡	0.2979
	CUDA	16.69	(no binning) + GPU	4.98917	0.211653‡	0.3000
(4)	Python	820	(no binning) + Parallelization	5.85 (1.66)§	–	–
	C++	days	(no binning) + Parallelization	5.677 (1.67)§	–	–
	CUDA	28.83	(no binning) + GPU	5.561 (1.60)§	–	–
(5)	CUDA	822.532	(no binning) + GPU	5.114 (1.14)§	0.2078	0.3021

† *Debyer* does not output time, but the simulations took less than 5 seconds to finish.

‡ The simulated value was 0.218335 × 10^2^.

§ Simulated value of 5.0 nm average value and 1.646 standard deviation.
