# Supplementary material for: Artificial intelligence in action: building simulation and analysis tools for powder diffraction
Source: Acta Crystallogr A Found Adv. 2025 Sep 4;81(Pt 5):401–4. doi: 10.1107/S2053273325007508 (PMC12415632; doi:10.1107/S2053273325007508)

Supporting Information: Artificial Intelligence in Action: Building Simulation and Analysis Tools for Powder Diffraction

P. Scardi & M.A. Malagutti, Department of Civil, Environmental and Mechanical Engineering, University of Trento, via Mesiano 77, 38123 Trento, Italy.

Contents

1 First acquaintance with ChatGPT: simulating a 6nm sphere with Python 2
2 Phase identification and Pawley fitting using Pseudo-Voigt for line profile 5
3 Whole Powder Pattern Modelling of 6 nm sphere 7
4 Microstrain emulation using python 10
5 Whole powder pattern modelling and Pair distribution function modelling in TOPAS 13
5.1 Whole powder pattern modelling 13
5.2 Double-Voigt approach form microstrain 14
5.3 Pair distribution function 15
6 Python code chatGPT prompts 15
6.1 Single sphere 15
6.2 Binned strategy for thermalization 15
6.3 Parallelization of microstrain calculation 15
6.4 Log-Normal crystallite size distribution 16
7 C++ codes chatGPT prompts 17
7.1 Single sphere 17
7.2 Binned strategy for thermallization 18
7.3 Parallelization of microstrain calculation 18
7.4 Log-Normal crystallite size distribution 18
8 Cuda codes chatGPT prompts 19
8.1 Single Sphere 19
8.2 Thermalization 19
8.3 Microstrain distribution 20
8.4 Crystallite size with microtrains distribution and thermal displacements 20
8.5 Summary of simulation times 21
9 Whole powder pattern modelling in TOPAS 21
9.1 TOPAS analysis: 21
9.1.1 Single Sphere 21
9.1.2 Thermalization 22
9.1.3 Parallelization of microstrain calculation 22
9.1.4 Log-Normal crystallite size distribution 23
9.2 Crystallite size, microstrain, and thermal displacements 24

1 First acquaintance with ChatGPT: simulating a 5 nm sphere with Python

Prompts necessary to arrive to the code below:

1- produce a plot with the simulation of a X-ray powder diffraction pattern from a powder made of identical spheres of Pd, about 6 nm diameter.
2- It seems to me that you did not use the atomic scattering factor of Pd. Please use it, and also add the Debye-Waller factor, with Debye-Waller coefficient of 0.4 Å^2
3- Why only two Bragg peaks? I'd expect to see others, all the way to 100 degrees
4- I think your calculation is wrong, intensity of peaks are too low, apart from the first two. Show me the expression you use for the atomic scattering factor
5- You used wrong coefficients for Pd in the Cromer–Mann parameterization, and be careful that coefficients you might find, most often, are given for s (2*sin(Theta)/lambda) in Å
6- The attached equation for f that you are using is wrong. The correct one is f = exp(- B * s^2)
7- You should use Multiplicity and a Lorentz-Polarization factor (actually polarization term could be omitted if we assume a fully polarized radiation, as from a synchrotron)
8- It looks wrong. It does not seem that you multiplied Intensities by the Multiplicity and Lorentz factor
9- Overlay experimental data and you'll see that your calculation is wrong
10- Your simulation is wrong. Double check all equations and write the expression you are using (in full)
11- s is sin(theta)/lambda. You are wrong by a factor of 2 Consider that if by s you mean the scattering vector modulus, then it's correct s = 2*sin/theta)/lambda. But in the expressions for atomict scattering factor and Debye-Waller factor, by s people mean sin(theta)/lambda, that is, half the scattering vector modulus
12- Why is (200) peak more intense than (111) in your simulation? There must be a mistake. Show me the euqation you are using with all terms. Find the error and correct it
13- Yes, the peak profile function should obviously be normalized to have unit area. Correct the simulation, shown me the result with the expt data, and show me again all equations
14- You are still wrong!!! You wrote that The (111) peak is now correctly dominant. But (200) it's higher than (111)
15- In the simulation you should include the Structure Factor (squared modulus). However, for Pd that would only multiply by a constant, so it's not the reaosn of your mistake. Please verify. However, plot all terms influencing the intensity as a function of 2theta
16- Calculate the exact integrated intensities
17- In your table above, for Multiplicity it's written 1.0 for (111) and 3.0 for (200) whi is wrong. And in fact, you wrote the correct Multiplicity on the summary (Key Results for Pd).

18- Now it looks correct. Explain me why you made the mistake, since the very beginning. You wrote the correct multiplicity while you were using wrong one in the simulation: how is it possible? Then, try using the alternative L factor, attached

19- The correct L factor should be 1/(sin(theta)*sin(thetaBragg)*cos(theta)) where thetaBragg is the theta angle for the Bragg condition, that is for the peak maximum

Python code:

```python
import numpy as np
import matplotlib.pyplot as plt
from scipy.spatial.distance import pdist
import sys

# ---------------------------------------------
# User-defined parameters
# ---------------------------------------------
a = 0.389                       # Pd lattice parameter in nm
wavelength = 0.154             # Cu Kα in nm
particle_diameter = 12.0       # Diameter of nanoparticle in nm
n_q_points = 300               # Number of q-values (300 is good for live plotting)
theta_2_min = 10               # Min 2-theta in degrees
theta_2_max = 90               # Max 2-theta in degrees
B_debye = 0.04                 # Debye-Waller factor in nm² (0.4 Å²)

# ---------------------------------------------
# Atomic scattering factor for Pd (Cromer-Mann in Å⁻¹ units)
# ---------------------------------------------
def atomic_scattering_factor(q_nm):
    # Convert q from nm⁻¹ to Å⁻¹
    q_angstrom = q_nm / 10.0
    s = q_angstrom / (4 * np.pi)
    a = [19.3319, 15.5017, 5.29537, 0.65844]
    b = [0.698655, 7.98929, 25.2052, 76.8968]
    c = 5.26593
    f = sum(a_i * np.exp(-b_i * s**2) for a_i, b_i in zip(a, b)) + c
    return f

# ---------------------------------------------
# Debye-Waller factor
# ---------------------------------------------
def debye_waller(q_nm, B=B_debye):
    return np.exp(-B * q_nm**2 / (16 * np.pi**2))

# ---------------------------------------------
# Generate FCC Pd nanoparticle
# ---------------------------------------------
basis = np.array([[0, 0, 0],
                  [0.5, 0.5, 0],
                  [0.5, 0, 0.5],
                  [0, 0.5, 0.5]])

n_cells = int(np.ceil(particle_diameter / a))
positions = []

for i in range(n_cells):
    for j in range(n_cells):
        for k in range(n_cells):
            cell_origin = np.array([i, j, k])
            for b in basis:
                pos = (cell_origin + b) * a
```

```
                if np.linalg.norm(pos - np.array([n_cells, n_cells, n_cells]) * a / 2)
<= particle_diameter / 2:
                    positions.append(pos)

positions = np.array(positions)
N_atoms = len(positions)
print(f"Generated {N_atoms} atoms for a ~{particle_diameter} nm Pd nanoparticle.\n")

# ---------------------------------------------
# Compute pairwise distances
# ---------------------------------------------
print("Calculating pairwise distances...")
distances = pdist(positions)

# ---------------------------------------------
# Scattering vector q and 2-theta values
# ---------------------------------------------
theta_2 = np.linspace(theta_2_min, theta_2_max, n_q_points)
theta = np.radians(theta_2 / 2)
q_vals = 4 * np.pi * np.sin(theta) / wavelength  # q in nm⁻¹

# ---------------------------------------------
# Set up live plotting
# ---------------------------------------------
plt.ion()
fig, ax = plt.subplots(figsize=(10, 6))
line, = ax.plot([], [], lw=1.5)
ax.set_xlim(theta_2_min, theta_2_max)
ax.set_ylim(0, 1.1)
ax.set_xlabel(r"2$\theta$ (degrees)")
ax.set_ylabel("Normalized Intensity (a.u.)")
ax.set_title(f"Simulated XRD Pattern (Pd, ~{particle_diameter} nm)")

# ---------------------------------------------
# Calculate intensity via Debye equation
# ---------------------------------------------
intensity = []
print("Calculating and plotting live:")
for idx, q in enumerate(q_vals):
    f_q = atomic_scattering_factor(q)       # uses q in Å⁻¹
    dwf = debye_waller(q)                   # q in nm⁻¹, B in nm²
    atomic_term = (f_q * dwf)**2

    sinc_vals = np.sinc((q * distances) / np.pi)
    I_q = atomic_term * (2 * np.sum(sinc_vals) + N_atoms)
    intensity.append(I_q)

    # Normalize and update plot
    I_norm = np.array(intensity)
    I_norm /= I_norm.max()
    line.set_data(theta_2[:idx+1], I_norm)
    ax.set_ylim(0, max(1.05, I_norm.max() * 1.1))
    fig.canvas.draw()
    fig.canvas.flush_events()
    plt.pause(0.001)  # required for Spyder GUI

    sys.stdout.write(f"\rProcessed {idx + 1}/{n_q_points} q-values")
    sys.stdout.flush()

print("\nCalculation complete.")
plt.ioff()
plt.show()
```

2 Phase identification and Pawley fitting using Pseudo-Voigt for line profile

Prompts used to do the phase identification:

1- I load a powder diffraction pattern, two columns: x (2theta in degrees), y (Intensity), collected using CuKalpha radiation, with lambda = 1.5406 Å. 1) plot the data, and carefully identify all Bragg peaks (no peaks below 39 degrees); then 2) show me a pattern fitting (use different colours for data end best fit) using Pawley method: refine the lattice parameter (it's a cubic structure, about 3.89 Å, but refine the best value), intensities and peak profile parameters of each Bragg peak using pseudo-Voigt functions (with different parameters for each peak). The background is not zero, so you might add a Chebyshev polynomial to the fit, using 3 or 4 coefficients 3) Can you guess what material could be? 4) estimate crystallite size 5) write a short report with formula used and all numerical results you obtained

2- Continuing the previous query, show me a Python code I can run in Spyder to obtain the Pawley fit above. The script should provide: (i) user control of starting parameters; (ii) a plot of data, model and their difference after convergence of least squares; (ii) a short report with the refined results

```
import numpy as np
import pandas as pd
import matplotlib.pyplot as plt
from scipy.optimize import curve_fit
from scipy.signal import find_peaks
from scipy.special import wofz

# --------------------------------
# USER SETTINGS
# --------------------------------
filename = 'Pd6nm3.xy'   # Path to your XY file
wavelength = 1.5406       # CuKalpha radiation
lattice_init = 3.89       # Initial guess for lattice parameter
eta_init = 0.5            # Initial pseudo-Voigt mixing
fwhm_init = 0.8           # Initial FWHM guess
K = 0.9                   # Scherrer constant

# --------------------------------
# Load and Prepare Data
# --------------------------------
df = pd.read_csv(filename, delim_whitespace=True, names=['2theta', 'Intensity'])
xdata = df['2theta'].values
ydata = df['Intensity'].values

# --------------------------------
# Bragg Peaks Detection (>39°)
# --------------------------------
```

```python
peaks, _ = find_peaks(ydata, height=max(ydata)*0.015, distance=6)
filtered_peaks = peaks[xdata[peaks] >= 39]
peak_positions = xdata[filtered_peaks]

# Tentative HKL assignments (FCC structure, filtered for 39°–130° range)
hkl_list = [(1,1,1), (2,0,0), (2,2,0), (3,1,1), (2,2,2),
            (4,0,0), (3,3,1), (4,2,0), (5,1,1)]

def d_to_2theta(d, wavelength):
    theta = np.arcsin(wavelength / (2*d))
    return np.degrees(2*theta)

# Validate HKLs
valid_hkls = []
for h, k, l in hkl_list:
    d = lattice_init / np.sqrt(h**2 + k**2 + l**2)
    if (wavelength / (2 * d)) <= 1:
        twotheta = d_to_2theta(d, wavelength)
        if 39 <= twotheta <= 130:
            valid_hkls.append((h, k, l))
hkl_list = valid_hkls

# -------------------------------
# Peak and Background Models
# -------------------------------
def pseudo_voigt(x, pos, eta, fwhm, scale):
    sigma = fwhm / (2 * np.sqrt(2 * np.log(2)))
    gamma = fwhm / 2
    z = ((x - pos) + 1j * gamma) / (sigma * np.sqrt(2))
    voigt = np.real(wofz(z)) / (sigma * np.sqrt(2*np.pi))
    return scale * ((1 - eta) * np.exp(-(x - pos)**2 / (2 * sigma**2)) + eta * voigt)

def chebyshev_background(x, c0, c1, c2, c3):
    x_scaled = 2 * (x - x.min()) / (x.max() - x.min()) - 1
    T0 = np.ones_like(x_scaled)
    T1 = x_scaled
    T2 = 2 * x_scaled**2 - 1
    T3 = 4 * x_scaled**3 - 3 * x_scaled
    return c0*T0 + c1*T1 + c2*T2 + c3*T3

def pawley_model(x, a, *params):
    n = len(hkl_list)
    eta_vals = params[:n]
    fwhm_vals = params[n:2*n]
    scale_vals = params[2*n:3*n]
    bg_coeffs = params[3*n:]
    ymodel = np.zeros_like(x)
    for i, (h, k, l) in enumerate(hkl_list):
        d = a / np.sqrt(h**2 + k**2 + l**2)
        twotheta = d_to_2theta(d, wavelength)
        ymodel += pseudo_voigt(x, twotheta, eta_vals[i], fwhm_vals[i], scale_vals[i])
    return ymodel + chebyshev_background(x, *bg_coeffs)

# -------------------------------
# Initial Parameters and Bounds
# -------------------------------
n_peaks = len(hkl_list)
init_params = [lattice_init] + [eta_init]*n_peaks + [fwhm_init]*n_peaks + \
              [max(ydata)/n_peaks]*n_peaks + [0, 0, 0, 0]
bounds_lower = [3.85] + [0]*n_peaks + [0.05]*n_peaks + [0]*n_peaks + [-np.inf]*4
bounds_upper = [3.95] + [1]*n_peaks + [2.0]*n_peaks + [np.inf]*n_peaks + [np.inf]*4

# -------------------------------
# Fit Pawley Model
```

```python
# ----------------------------------
popt, _ = curve_fit(pawley_model, xdata, ydata, p0=init_params,
                    bounds=(bounds_lower, bounds_upper), maxfev=15000)

a_refined = popt[0]
eta_vals = popt[1:n_peaks+1]
fwhm_vals = popt[n_peaks+1:2*n_peaks+1]
scale_vals = popt[2*n_peaks+1:3*n_peaks+1]
bg_coeffs = popt[3*n_peaks+1:]
yfit = pawley_model(xdata, *popt)

# ----------------------------------
# Scherrer Crystallite Size
# ----------------------------------
d_111 = a_refined / np.sqrt(3)
theta_111 = d_to_2theta(d_111, wavelength) / 2
theta_rad = np.radians(theta_111)
fwhm_rad = np.radians(fwhm_vals[0])
D_angstrom = (K * wavelength) / (fwhm_rad * np.cos(theta_rad))
D_nm = D_angstrom / 10

# ----------------------------------
# Plot: Data, Fit, Difference
# ----------------------------------
plt.figure(figsize=(10, 6))
plt.plot(xdata, ydata, label='Experimental', color='black')
plt.plot(xdata, yfit, label='Pawley Fit', color='red', linestyle='--')
plt.plot(xdata, ydata - yfit, label='Residual', color='blue')
plt.xlabel(r"2$\theta$ (degrees)")
plt.ylabel("Intensity (a.u.)")
plt.title("Pawley Refinement Fit")
plt.legend()
plt.grid(True)
plt.tight_layout()
plt.show()

# ----------------------------------
# Rwp
# ----------------------------------
residuals = ydata - yfit
Rwp = np.sqrt(np.sum(residuals**2) / np.sum(ydata**2)) * 100

# ----------------------------------
# Output: Short Report
# ----------------------------------
print("\n--- Pawley Refinement Results ---")
print(f"Lattice parameter a: {a_refined:.4f} Å")
print(f"Crystallite size from (111): {D_nm:.2f} nm")
print(f"Rwp (fit quality): {Rwp:.2f} %")
print("\nPeak Parameters (η, FWHM, Intensity):")
for i, (eta, fwhm, scale) in enumerate(zip(eta_vals, fwhm_vals, scale_vals)):
    print(f"  Peak {i+1}: η={eta:.2f}, FWHM={fwhm:.2f}°, Intensity={scale:.2f}")
print("\nChebyshev Background Coefficients:")
print(f"  c0={bg_coeffs[0]:.4f}, c1={bg_coeffs[1]:.4f}, c2={bg_coeffs[2]:.4f},
c3={bg_coeffs[3]:.4f}")
```

3 Whole Powder Pattern Modelling of 6 nm sphere

1- Attached is the simulated X-ray scattering pattern of a Pd nanosphere of 6 nm in diameter. Make a python code that performs a Pawley fit to this data. It should have the lattice

parameter and diameter as refinable variables, and the peak intensities should be freely refined. Graph the results of given pattern, modelled pattern, and residuals. Output the final lattice parameters and diameter in Angstroms. The wavelength of the pattern is lambda = 1.5406 Angstroms. To perform the fit of the line profile, use the expression attached, where D is the diameter, d* = 2 sin(theta)/lambda, and d*hkl is the peak center position from the Bragg Law. Output the equation used. Output the code in the ChatGPT prompt

2- The code works. Edit the code by adding more diffraction peaks to the pattern to fit all the pattern 2theta range. In addition, output the value of the refined lattice parameter and diameter in the prompt, together with the quality of fit. Keep outputing the pattern, the modelled profiled, and residuals. Do not perform the fit internally, just alter the code.

```
import numpy as np
import pandas as pd
import matplotlib.pyplot as plt
from scipy.optimize import curve_fit

# --------------------------------
# USER SETTINGS
# --------------------------------
filename = 'Pd6nm3.xy'   # Update this to match your data path
wavelength = 1.5406       # CuKalpha (Å)
a_init = 3.89             # Initial lattice parameter guess (Å)
D_init = 100              # Initial crystallite diameter (Å)

# --------------------------------
# Load and Prepare Data
# --------------------------------
df = pd.read_csv(filename, delim_whitespace=True, names=['2theta', 'Intensity'])
xdata = df['2theta'].values
ydata = df['Intensity'].values

# Bragg peak assignments (from earlier detection)
hkl_list = [(1,1,1), (2,0,0), (2,2,0), (3,1,1), (2,2,2),
            (4,0,0), (3,3,1), (4,2,0), (5,1,1)]

# --------------------------------
# Model Components
# --------------------------------
def d_to_2theta(d, wavelength):
    theta = np.arcsin(wavelength / (2*d))
    return np.degrees(2*theta)

def twotheta_to_s(two_theta, wavelength):
    theta = np.radians(two_theta / 2)
    return 2 * np.sin(theta) / wavelength

def spherical_peak_profile(x, s0, D, scale):
    s = twotheta_to_s(x, wavelength)
    delta = np.pi * D * (s - s0)
    profile = np.zeros_like(x)
    mask = delta != 0
    profile[mask] = scale / (8 * delta[mask]**4) * (
        3 * D * (1 + 2 * delta[mask]**2 - np.cos(2*delta[mask]) -
2*delta[mask]*np.sin(2*delta[mask]))
    )
```

```python
    profile[~mask] = 3 * D / 4  # limiting value at delta=0
    return profile

def chebyshev_background(x, c0, c1, c2, c3):
    x_scaled = 2 * (x - x.min()) / (x.max() - x.min()) - 1
    T0 = np.ones_like(x_scaled)
    T1 = x_scaled
    T2 = 2 * x_scaled**2 - 1
    T3 = 4 * x_scaled**3 - 3 * x_scaled
    return c0*T0 + c1*T1 + c2*T2 + c3*T3

# -------------------------------
# Full Model
# -------------------------------
def model(x, a, D, *params):
    n = len(hkl_list)
    scale_vals = params[:n]
    bg_coeffs = params[n:]
    ymodel = np.zeros_like(x)
    for i, (h, k, l) in enumerate(hkl_list):
        d = a / np.sqrt(h**2 + k**2 + l**2)
        s0 = 1 / d
        ymodel += spherical_peak_profile(x, s0, D, scale_vals[i])
    return ymodel + chebyshev_background(x, *bg_coeffs)

# -------------------------------
# Initial Guess and Fitting
# -------------------------------
n_peaks = len(hkl_list)
init_scales = [max(ydata)/n_peaks] * n_peaks
init_bg = [0, 0, 0, 0]
init_params = [a_init, D_init] + init_scales + init_bg

lower_bounds = [3.85, 10] + [0]*n_peaks + [-np.inf]*4
upper_bounds = [3.95, 1000] + [np.inf]*n_peaks + [np.inf]*4

# Plot initial guess
y_guess = model(xdata, *init_params)
plt.figure(figsize=(10, 6))
plt.plot(xdata, ydata, label='Experimental', color='black')
plt.plot(xdata, y_guess, label='Initial Guess', color='orange', linestyle='--')
plt.title("Initial Guess Before Refinement")
plt.xlabel(r"2$\theta$ (degrees)")
plt.ylabel("Intensity (a.u.)")
plt.legend()
plt.grid(True)
plt.tight_layout()
plt.show()

# Perform least-squares fit
popt, _ = curve_fit(model, xdata, ydata, p0=init_params,
                    bounds=(lower_bounds, upper_bounds), maxfev=20000)

# Extract parameters
a_refined = popt[0]
D_refined = popt[1]
scale_vals = popt[2:2+n_peaks]
bg_coeffs = popt[-4:]
yfit = model(xdata, *popt)

# -------------------------------
# Plot Final Fit
# -------------------------------
plt.figure(figsize=(10, 6))
```

```
plt.plot(xdata, ydata, label='Experimental', color='black')
plt.plot(xdata, yfit, label='Refined Fit', color='red', linestyle='--')
plt.plot(xdata, ydata - yfit, label='Residuals', color='blue')
plt.xlabel(r"2$\theta$ (degrees)")
plt.ylabel("Intensity (a.u.)")
plt.title("Refined Spherical Profile Pawley Fit")
plt.legend()
plt.grid(True)
plt.tight_layout()
plt.show()

# -------------------------------
# Report Final Results
# -------------------------------
print("\n--- Spherical Profile Pawley Fit Report ---")
print(f"Refined lattice parameter a: {a_refined:.4f} Å")
print(f"Refined crystallite size D: {D_refined:.2f} Å")
print("\nChebyshev background coefficients:")
print(f"  c0 = {bg_coeffs[0]:.4e}")
print(f"  c1 = {bg_coeffs[1]:.4e}")
print(f"  c2 = {bg_coeffs[2]:.4e}")
print(f"  c3 = {bg_coeffs[3]:.4e}")
print("\nPeak intensities:")
for i, (hkl, scale) in enumerate(zip(hkl_list, scale_vals)):
    print(f"  Peak {i+1} ({hkl}): Scale = {scale:.2f}")
```

4 Microstrain emulation using python

This script considers the atoms as independent (Einstein) oscillators, so it uses a Gaussian distribution of atomic displacements in the three spatial directions, such that the total mean square displacement (MSD) is $MSD = B_{iso}/8\pi^2$, where B_{iso} is the isotropic DW coefficient. *n* configurations were emulated, with corresponding powder patterns generated with the DSE summed for better randomization. The microstrain model implemented in the ChatGPT simulations involved generating 30 nanospheres of similar diameter (called bins), each with a slightly different lattice parameter. The Debye scattering equation was calculated for each of these spheres, being multiplied by the gaussian weights of a normal distribution having 3.89 Å as average value and 1% variance (see Figure S 1 for visual representation). This approach simulates chemical microstrain, so named because each crystallite is assumed to exhibit slight chemical variations that induce isotropic expansions or contractions of the lattice parameter.

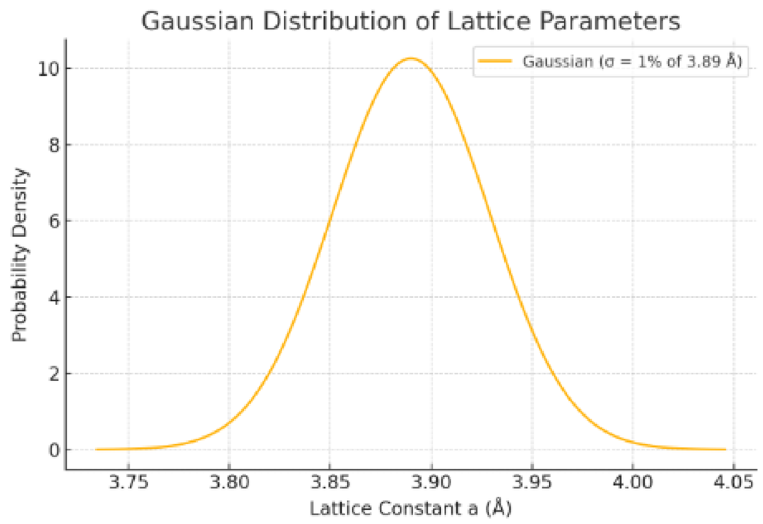

Figure S 1. Probability density of a normal distribution of lattice parameters used to emulate a chemical microstrain effect.

These are the prompt used to generate the code below:

1-With the code shown below one can simulate the diffraction pattern from a powder made of small identical nanocrystals of spherical shape. A Debye-Waller factor has been added to include thermal effects. Create a new code without using a Debye-Waller factor. Instead, add random displacement to all atomic positions and then calculate the powder pattern. In doing this: allow me to change the Debye-Waller coefficient (equal to 8*PI^2*MSD, with MSD=mean square displacement) at pleasure; consider that random displacement must be added separately for x, y and z coordinates of each atom, according to a Gaussian distribution for each coordinates, such that total MSD is three times the MSD along one coordinate; add the possibility to average over a chosen number of random configurations to improve the statistics; allow me to switch on/off the live plotting; use Angstrom units.

The following code includes: (i) input data (cell parameter, wavelength, nanocrystal diameter, range and number of points sampled in reciprocal space ($q = 4\pi \sin\theta / \lambda$), and DW coefficient), (ii) calculation of f and DW factor, (iii) generation of atomic coordinates inside the Pd sphere of given diameter, (iv) calculation of all atom pair distances, (v) calculation of the diffracted intensity by Debye Scattering Equation (DSE), and finally (vi) a live plotting and/or data output routine.

```
import numpy as np
import matplotlib.pyplot as plt
from scipy.spatial.distance import pdist
from scipy.stats import norm
import sys

# ----------------------------------------------
# User-defined parameters
# ----------------------------------------------
a_mean = 3.89                          # Mean lattice parameter in Å
a_std_percent = 1.0                   # Standard deviation as % of a_mean
n_lattice_samples = 20                # Number of lattice parameter samples
wavelength = 1.54                     # Cu Kα in Å
particle_diameter = 100.0             # Nanocrystal diameter in Å
n_q_points = 300
```

```python
theta_2_min = 10
theta_2_max = 90
B_coefficient = 0.4               # Debye-Waller B coefficient in Å²
n_configurations = 5              # Random configs per lattice
live_plotting = False
save_file = "simulated_pattern.xy"

# ---------------------------------------------
# Atomic scattering factor for Pd
# ---------------------------------------------
def atomic_scattering_factor(q_angstrom):
    s = q_angstrom / (4 * np.pi)
    a = [19.3319, 15.5017, 5.29537, 0.65844]
    b = [0.698655, 7.98929, 25.2052, 76.8968]
    c = 5.26593
    f = sum(a_i * np.exp(-b_i * s**2) for a_i, b_i in zip(a, b)) + c
    return f

# ---------------------------------------------
# Generate FCC Pd nanoparticle
# ---------------------------------------------
def generate_positions(a, diameter):
    basis = np.array([[0, 0, 0],
                      [0.5, 0.5, 0],
                      [0.5, 0, 0.5],
                      [0, 0.5, 0.5]])
    n_cells = int(np.ceil(diameter / a))
    positions = []

    for i in range(n_cells):
        for j in range(n_cells):
            for k in range(n_cells):
                cell_origin = np.array([i, j, k])
                for b in basis:
                    pos = (cell_origin + b) * a
                    if np.linalg.norm(pos - np.array([n_cells, n_cells, n_cells]) * a
/ 2) <= diameter / 2:
                        positions.append(pos)
    return np.array(positions)

# ---------------------------------------------
# Simulate powder pattern for one configuration
# ---------------------------------------------
def simulate_pattern(positions, q_vals, std_dev, n_config):
    N_atoms = len(positions)
    intensity_avg = np.zeros_like(q_vals)

    for _ in range(n_config):
        displaced = positions + np.random.normal(scale=std_dev, size=positions.shape)
        distances = pdist(displaced)
        intensity = []

        for q in q_vals:
            f_q = atomic_scattering_factor(q)
            sinc_vals = np.sinc((q * distances) / np.pi)
            I_q = f_q**2 * (2 * np.sum(sinc_vals) + N_atoms)
            intensity.append(I_q)

        intensity_avg += np.array(intensity)

    return intensity_avg / n_config

# ---------------------------------------------
# Main Simulation
```

```
# ---------------------------------------------
theta_2 = np.linspace(theta_2_min, theta_2_max, n_q_points)
theta = np.radians(theta_2 / 2)
q_vals = 4 * np.pi * np.sin(theta) / wavelength  # q in Å⁻¹

# Correct standard deviation from B for one direction
std_displacement = np.sqrt(B_coefficient / (8 * np.pi**2))

# Lattice parameter sampling
if a_std_percent == 0:
    lattice_constants = [a_mean]
    weights = [1.0]
else:
    a_std = a_mean * a_std_percent / 100
    lattice_constants = np.linspace(a_mean - 3*a_std, a_mean + 3*a_std,
n_lattice_samples)
    weights = norm.pdf(lattice_constants, loc=a_mean, scale=a_std)
    weights /= np.sum(weights)  # Normalize

intensity_sum = np.zeros_like(q_vals)

print("Starting powder pattern simulation...")
for i, (a, w) in enumerate(zip(lattice_constants, weights)):
    print(f"  Lattice {i+1}/{len(lattice_constants)}: a = {a:.5f} Å, weight =
{w:.4f}")
    pos = generate_positions(a, particle_diameter)
    I = simulate_pattern(pos, q_vals, std_displacement, n_configurations)
    intensity_sum += w * I

intensity_sum /= np.max(intensity_sum)  # Normalize

# ---------------------------------------------
# Plot and Save
# ---------------------------------------------
plt.figure(figsize=(10, 6))
plt.plot(q_vals, intensity_sum, lw=1.5)
plt.xlabel("q (Å⁻¹)")
plt.ylabel("Normalized Intensity (a.u.)")
plt.title("Simulated Powder Pattern (Pd)")
plt.grid(True)
plt.tight_layout()
plt.show()

np.savetxt(save_file, np.column_stack((q_vals, intensity_sum)), fmt="%.6f",
header="q(Å⁻¹) Intensity", comments='')
print(f"\n✅ Final powder pattern saved to '{save_file}'")
```

5 Whole powder pattern modelling and Pair distribution function modelling in TOPAS

5.1 Whole powder pattern modelling

The reciprocal space data fitting employed WPPM and Double-Voigt (DV) approaches to model crystallite size and microstrain contributions, respectively. WPPM relies on equations for crystallite shape and size distribution retrieved after Fourier transformation (FT) of the line profile

contributions (Jagodzinski, 1963). For a single sphere, the FT of the line profile due to the crystallite size contribution is (Scardi *et al.*, 2018):

$$A^S(L) = q_0(L) - \frac{3}{2}q_1(L) + \frac{1}{2}q_3(L)$$

This is achieved in TOPAS using the following macro:

```
macro WPPM_Sphere(RRc, RRv)
   {
      If_Prm_Eqn_Rpt(RRc, RRv, min .1 max = Min(2 Val + .3, 10000);)
      WPPM_ft_conv = 1 - 0.75*WPPM_L/RRv + 0.0625*(WPPM_L/RRv)^3 ;

      'WPPM_break_on_small = 1e-7;
      WPPM_L_max = 2*CeV(RRc, RRv);
      WPPM_th2_range = 55;
   }
```

5.2 Double-Voigt approach form microstrain

A gaussian profile function with tangent 2θ dependence [vide (Wilson, 1949)] can be employed for microstrain modelling using the following instructions in TOPAS:

```
prm E0G  0.00511719757`
prm !arg_E0G = 360/Pi E0G;:0.586387647`
Strain_G( arg_E0G )
prm !E0 = Voigt_FWHM_GL(E0G, 0);:0.00511719757` ' fwhm of the microstrain distribution
prm !deltaG = E0 / (2 Sqrt(2 Ln(2)));:0.00217307373` ' standard deviation of the microstrain distribution
```

where a gaussian function defined as:

$$\text{gauss}(2\theta) = \frac{2\sqrt{\ln(2)/\pi}}{\text{fwhm}} \; e^{-4\ln(2)\left(\frac{2\theta}{\text{fwhm}}\right)^2}$$

is convolved with the crystallite size contribution. Here $fwhm = e_0\,\text{Tan}(\theta)$ is the full-width at half maximum of the peak with e_0 the full-width at half maximum of the microstrain distribution. See details in (Dinnebier *et al.*, 2018).

5.3 Pair distribution function

PDF was calculated using PDFGetX3 (Juhás *et al.*, 2013) from the simulated reciprocal space data, employing analytical expressions for spherical shape functions for size modelling and gaussian peak profiles for dynamic (DW) and static (microstrain) contributions as described elsewhere (Beyer *et al.*, 2022; Thorpe *et al.*, 2002).

6 Python code chatGPT prompts

The strategies employed in (1) brute force calculation of DSE for a single 5 nm sphere of fcc Pd, (2) binning the pair distances and calculation of thermal Einstein model, (3) Parallelization of the code, distribution the calculation of each microstrain step to a single thread, (4) simulation a log normal distribution of spheres with parallelization strategy, and (5) using cuda language to simulate a size distribution of spheres, containing microstrain and thermal effects altogether.

6.1 Single sphere

Prompt:

Create a python code that creates spherical Pd nanoparticles given the lattice parameter of an fcc structure. One should be able to choose the diameter. Then proceed with the calculation of the debye scattering equation, where the minimum, maximum and step of 2theta can be chosen as hard coded variables. Do not calculate the distance between i and j atoms twice. Allow to change the wavelength of the radiation. Use form factors from literature. use Angstrom units. Save the I(2theta) plot in a two column file with .xy extension. Tell the progress of the calculation in the prompt Add computational time calculation in minutes. Output the code in prompt.

6.2 Binned strategy for thermalization

Prompt:

From the previous code, add thermal displacements to the atoms by randomly shifting the atoms from their original positions in the X, Y, Z directions independently. This shift should be weighted on a gaussian function with variance equal to the mean square displacement (MSD) = 3/(8 Pi^2) Biso, where Biso is the isotropic Debye-Waller factor. Let me tune the number of times this randomisation is performed. Perform the binning of the pair distances and allow to choose the bin size. Inform about the progress for each calculation, showing how many bins are left to calculate. Average the intensities obtained for each randomised sphere. Output the code here in the prompt.

6.3 Parallelization of microstrain calculation

Prompt:

Upgrade the same code to generate several nanospheres with varying lattice parameters centered in an average value, distributed 0.15 Angstrons for each side, with a fwhm of the gaussian distribution of 0.01 Angstroms. Calculate a total of 20 different lattice parameter steps. For each lattice parameter, perform the simulation of thermal effects and average the scattering pattern using the the same Biso. Set the number of lattice parameter steps as a fixed variable. Calculate the Debye scattering equation, placing a lattice step for each process and calculating them independently from each other. Allow to choose the number of process as a fixed variable. After computing the Debye scattering equation for each lattice step, multiply the intensity by its gaussian weight, sum the contribution for each lattice parameter step and output an .xy file for the weighted pattern, emulating the distribution of microstrain. Inform in the prompt how many bins are calculated in a 10000 interval, which thread is calculating, and which random displacement step is being performed. output the code in chatgpt terminal.

6.4 Log-Normal crystallite size distribution

upgrade the previous code to simulate a log-normal distribution of spheres. Calculate the Debye scattering equation for sphere diameters from 0 to 10 nm, with intervals of 0.389 nm. Use maps to perform the binning, avoiding to store bins with zeroes. Empty the map after completing the calculation for each sphere. Use float 32 precision. For the parallelisation, associate one crystallite size calculation with a process. Output the scattering pattern for each sphere in a .xy file. At the end, multiply the intensity of each .xy by its log-normal weight, sum the intensity of all .xy files, normalise it, and output the result in a separate .xy file. Allow to change the log-normal mean and standard deviation. Calculate the total time of calculation, continue outputting the information on which sphere is being calculated how many non zero bins are remaining. Do not perform thermalization or microstrian calculation. Perform a solution to use less ram memory by zeroing the maps after use. Use these values as inputs:

mean_lattice_param = 3.89   # Angstrom

num_steps = 20   # Total lattice parameter steps

num_processes = 20   # Number of parallel processes

diameter = 50.0   # Nanoparticle diameter

wavelength = 0.3   # Angstrom

Biso = 0.3   # Debye-Waller factor

n_randomisations = 5   # Random displacements per lattice

bin_size = 0.001   # Bin size in Angstrom

theta_min = 0.01

theta_max = 160.0

theta_step = 0.01

*MSD = 1 / (8 * np.pi**2) * Biso*

a = [19.3319, 0.698655 , 15.5017, 7.98929, 5.29537 , 25.2052, 0.605844, 76.8986]

c = 5.26593

average_Size = 5nm #nm

standard_deviation = 0.6

2- how can we eliminate the problem of overloading the memory?

Answer:

✅ Summary: Safe, Efficient, and Extensible

Strategy	In Use?	Benefit
Use of `float32`	✅	50% less memory
Skip empty bins (map)	✅	Avoids huge empty arrays
Clear bins after use	✅	Releases memory immediately
One sphere per process	✅	Keeps each process isolated
Running average / counts only	⬜	Optional, for even less memory
On-disk intermediate storage	⬜	Optional, for huge datasets

7 C++ codes chatGPT prompts

7.1 Single sphere

Prompt:

Create a c++ code that creates spherical Pd nanoparticles given the lattice parameter of an fcc structure. One should be able to choose the diameter. Then proceed with the calculation of the debye scattering equation, where the minimum, maximum and step of 2theta can be chosen as hard coded variables. Do not calculate the distance between i and j atoms twice. Allow to change the wavelength of the radiation. Use form factors from literature. use Angstrom units. Save the I(2theta) plot in a two column file with .xy extension. Tell the progress of the calculation in the prompt Add computational time calculation in minutes. Output the code in prompt.

7.2 Binned strategy for thermallization

Prompt:

From the previous code, add thermal displacements to the atoms by randomly shifting the atoms from their original positions in the X, Y, Z directions independently. This shift should be weighted on a gaussian function with variance equal to the mean square displacement (MSD) = 1/(8 Pi^2) Biso, where Biso is the isotropic Debye-Waller factor. Let me tune the number of times this randomisation is performed. Perform the binning of the pair distances and allow to choose the bin size. Inform about the progress for each calculation, showing how many bins are left to calculate. Average the intensities obtained for each randomised sphere. Output the code here in the prompt.

7.3 Parallelization of microstrain calculation

Prompt:

Now, optimise the same code to simulate several nanospheres with a distribution of lattice parameters with variance corresponding to that of a gaussian distribution. The simulation of thermal effects must be done for each lattice parameter calculated. Allow me to change the number of lattice parameter steps. Perform a parallel calculation of the Debye scattering equation, where each lattice step is associated with a single thread. After computing the Debye scattering equation for each lattice step, multiply the intensity by its gaussian weight, normalise it, and output an .xy file for the weighted pattern. Inform in the prompt how many bins are calculated in a 10000 interval, which thread is calculating, and which random displacement step is being performed. Output a solution for visual studio code. output the code in chatgpt terminal.

7.4 Log-Normal crystallite size distribution

Prompt:

Create a new code to simulate a log-normal distribution of spheres based on the functions defined in the previous step. Calculate the Debye scattering equation for sphere diameters from 0 to 10 nm, with intervals of 0.389 nm. Do not employ a binning strategy. Do a double parallelisation, dividing the work on each crystallite size step and associating the calculation with a range of 2theta to a single thread. Optimise this parallelisation for a 24-thread system. Output the scattering pattern for each sphere in a .xy file after it finishes, so we can check if the simulations are working well. At the end, multiply the intensity of each .xy by its normalised log-normal weight, sum the intensity of all .xy files, and output the result in a separate .xy file. Allow to change the log-normal

mean and standard deviation. Calculate the total time of calculation, and continue outputting the information on which sphere is being calculated. Do not perform thermalisation or microstrain calculation. Use these values as inputs: mean_lattice_param = 3.89 # Angstrom num_processes = 20 # Number of parallel processes wavelength = 0.3 # Angstrom theta_min = 0.01 theta_max = 160.0 theta_step = 0.01 form factor a = [19.3319, 0.698655 , 15.5017, 7.98929, 5.29537 , 25.2052, 0.605844, 76.8986] c = 5.26593 average_Size = 5nm #nm standard_deviation = 0.6 Cuda codes chatGPT prompts.

Please check if the units are correct (between Angstroms and nm). The code is not calculating the patterns very well. Modify the code and output here in the prompt being sure that: 1) It generates a sphere of diameter D 2) Calculates the Debye scattering equation for the sphere D (parallelising the calculation by dividing the 2theta region into 24 threads). 3) Outputs the DSE pattern for each D sphere 4) At the end, read the DSE pattern for each sphere. Multiply the intensity by its normalised log-normal distribution given mu and sigma. Sum the intensity of all the DSE patterns. Please, use the same functions as in the previous code to generate the patterns and calculate the DSE.

8 Cuda codes chatGPT prompts

8.1 Single Sphere

Prompt:

*Create a cuda code that creates spherical Pd nanoparticles given the lattice parameter of an fcc structure. One should be able to choose the diameter. Then proceed with the calculation of the debye scattering equation, where the minimum, maximum and step of 2theta can be chosen as hard coded variables. Do not calculate the distance between i and j atoms twice. Allow to change the wavelength of the radiation. Use a 9 coefficient form factor> def form_factor(Q): a = form_factor_coeffs Q_scaled = (Q / (4 * np.pi))**2 f = (a[0]*np.exp(-a[1]*Q_scaled) + a[2]*np.exp(-a[3]*Q_scaled) + a[4]*np.exp(-a[5]*Q_scaled) + a[6]*np.exp(-a[7]*Q_scaled) + c) return f.astype(np.float32) form_factor_coeffs = [19.3319, 0.698655, 15.5017, 7.98929, 5.29537, 25.2052, 0.605844, 76.8986] c = 5.26593. use Angstrom units. Save the I(2theta) plot in a two column file with .xy extension. Tell the progress of the calculation in the prompt Add computational time calculation in minutes. Output the code in prompt. Make a code adapted to NVIDIA Corporation GK110 [GeForce GTX TITAN] graphic card, 15 Gb of ram*

8.2 Thermalization

Prompt1:

From the previous code, add thermal displacements to the atoms by randomly shifting the atoms from their original positions in the X, Y, Z directions independently. This shift should be weighted on a gaussian function with variance equal to the mean square displacement (MSD) = 1/(8 Pi^2) Biso, where Biso is the isotropic Debye-Waller factor. Let me tune the number of times this randomisation is performed. Average the intensities obtained for each randomised sphere. Output the code here in the prompt.

Prompt2:

The previous code presented zero values for intensities. To debug, output the scattering pattern for each ramdomized particle

8.3 Microstrain distribution

Prompt:

Upgrade the same code to generate several nanospheres with varying lattice parameters centered in an average value, distributed 0.15 Angstrons for each side, with a fwhm of the gaussian distribution of 0.01 Angstroms.Calculate a total of 20 different lattice parameter steps. This can be achieved by looping through generate_fcc_sphere function with different lattice parameters. For each lattice parameter, perform the simulation of thermal effects and average the scattering pattern using the the same Biso by looping thorough the computeDebyeWithThermalAveraging function. After computing the Debye scattering equation for each lattice step, multiply the intensity by its gaussian microstrain weight, sum the contribution for each lattice parameter step, normalized it, and output an .xy file for the weighted pattern, emulating the distribution of microstrain. Inform the progress of the displacements and lattice step calculations in the prompt. output the code in chatgpt terminal.

Several prompts were necessary to fix bugs:

I need to reduce the parameters too much. Lets go with option 2, by splitting the debye scattering equation calculation into several chuncks. What do you recommend for the graphic card in my pc?

The code runs and the results look approximatelly good, showing the bragg peaks, the small angle scattering contribution, and thermal diffuse components. The problem is that we find negative intensities as if the pattern was shifted to lower intensities. What do you suspect could be the problem?

The prompts above were necessary to correct for overloading the GPU and silent errors.

8.4 Crystallite size with microtrains distribution and thermal displacements

Prompt:

Now let's use the same functions in order to simulate a log-normal distribution of spheres with thermalization and microstrain included. Employ the same functions developed previously. Loop the

creation of spheres with the same average lattice parameter, add the microstrain steps in the range of 0.015 Angstroms and FWHM = 0.01 Angstroms, add thermalization for each microstrain step. For each microstrain step, average the pattern by the number of thermalizations performed.Make a weighted sum of all the patterns for each microstrain step, using a normalized gaussian function. For each diameter step, multiply the scattering pattern by its normalized log-normal weight and sum all the patterns, outputting the final pattern with crystallite size distribution, thermalization, and microstrain. Output the .xy scattering pattern for each sphere step (after themalization and microstrain emulation) and the final crystallite size distribution output. Output the entire code here in the prompt.

8.5 Summary of simulation times

9 Whole powder pattern modelling in TOPAS

9.1 TOPAS analysis:

9.1.1 Single Sphere

Python

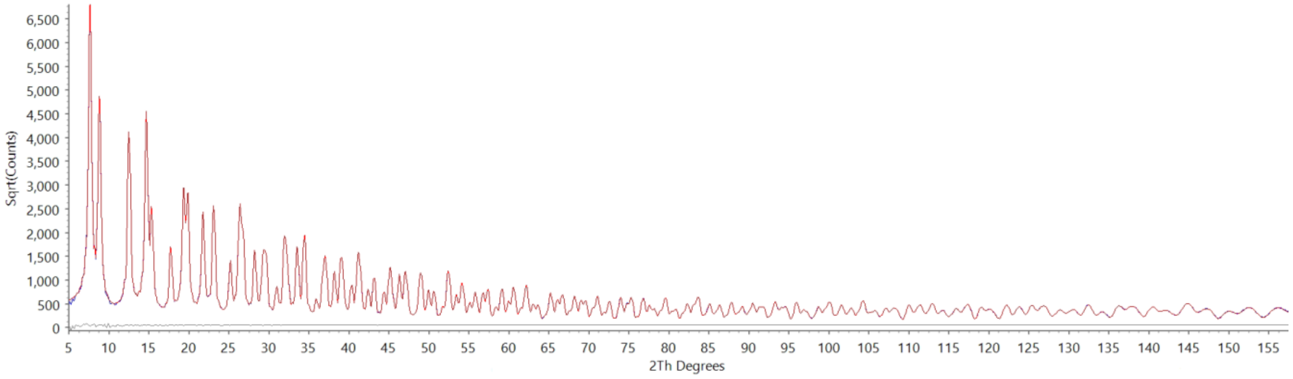

C++ code

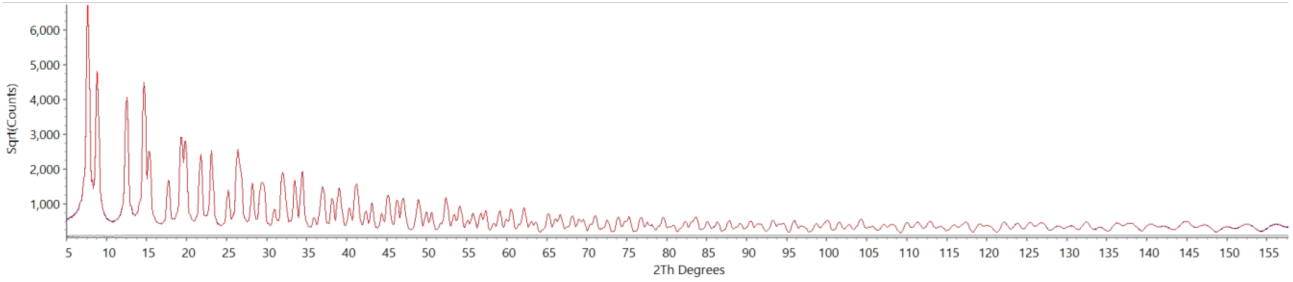

Cuda:

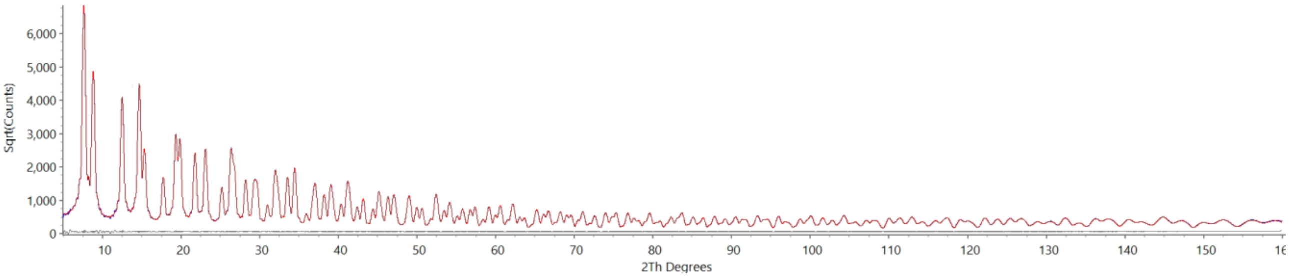

9.1.2 Thermalization

Python

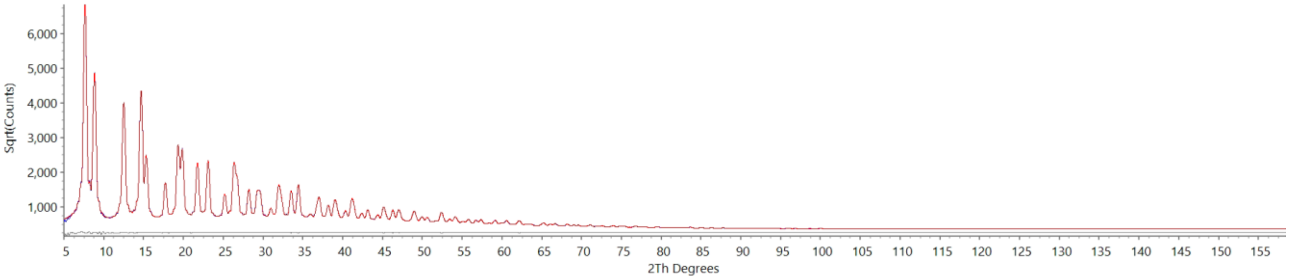

C++

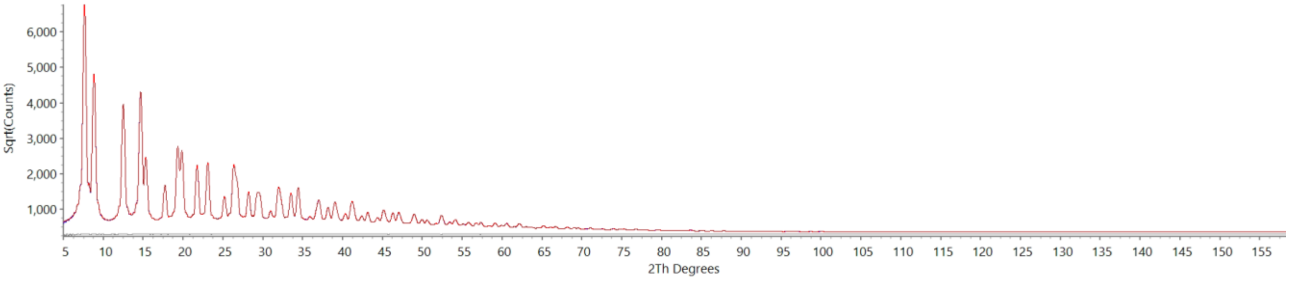

Cuda

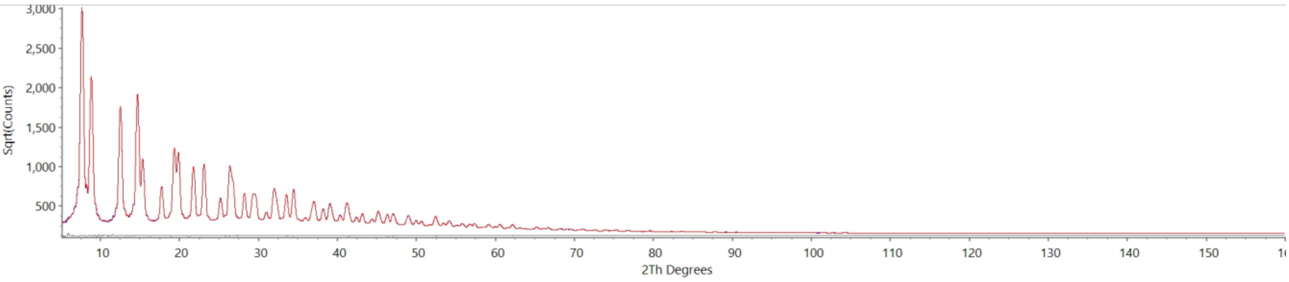

9.1.3 Parallelization of microstrain calculation

Python

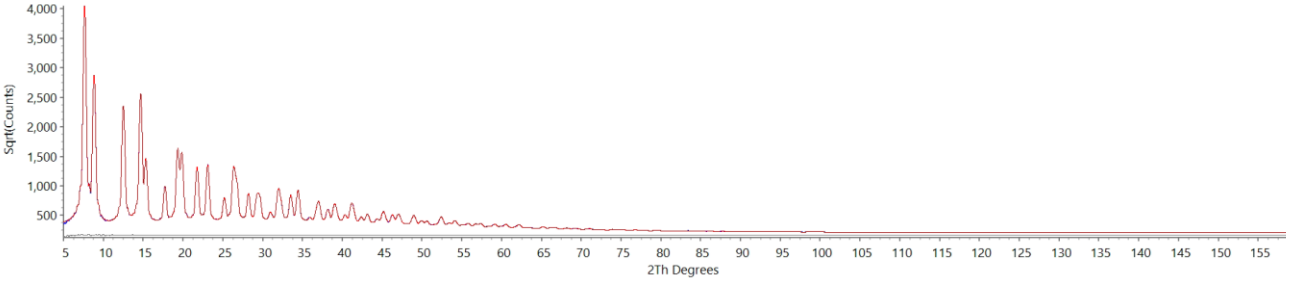

C++

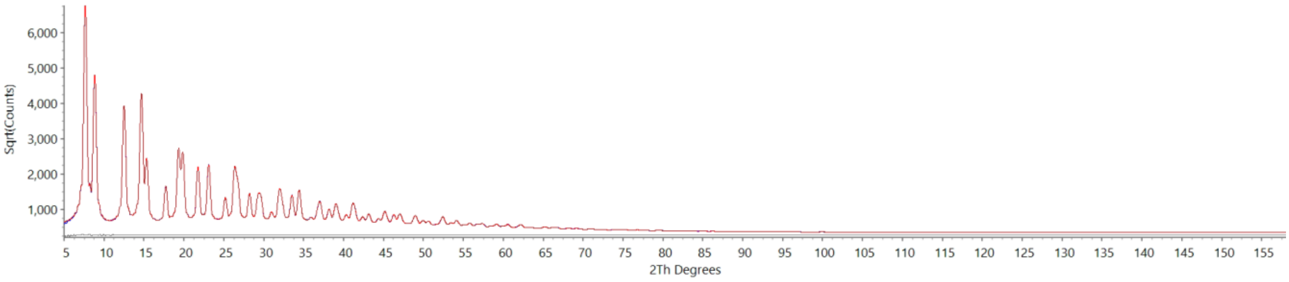

Cuda:

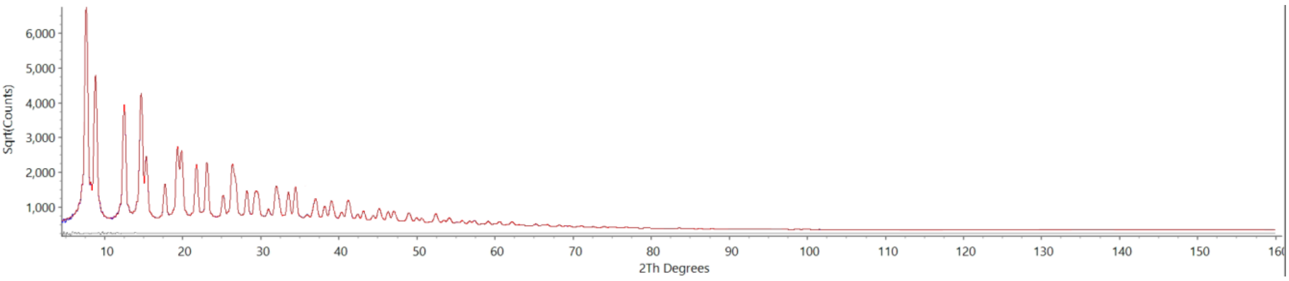

9.1.4 Log-Normal crystallite size distribution

Python:

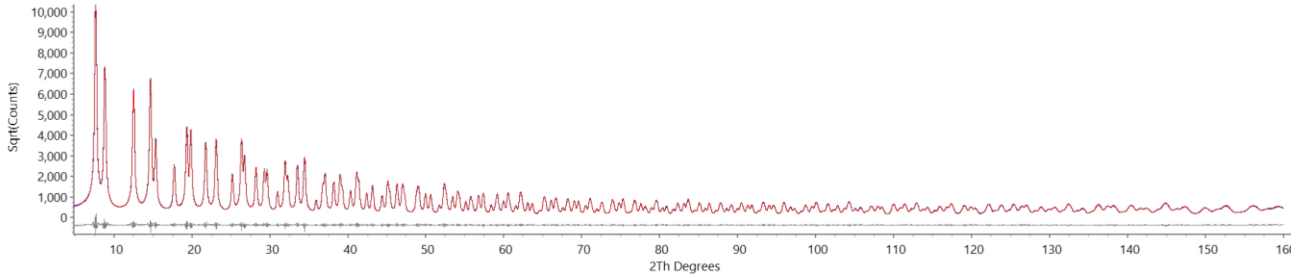

C++:

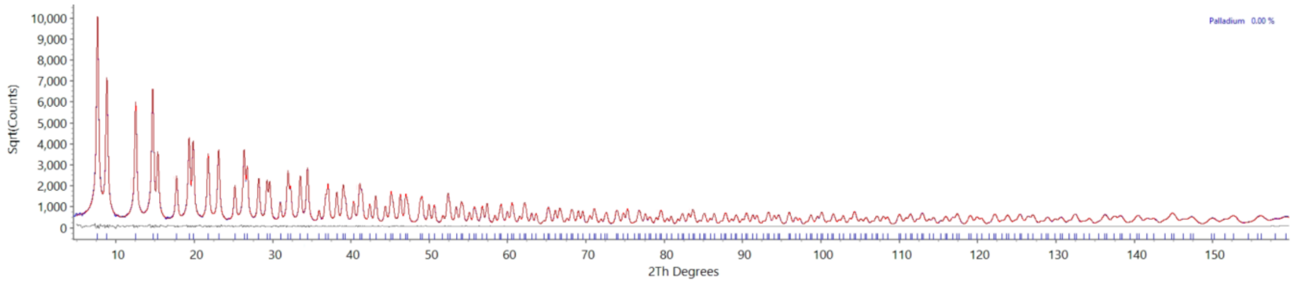

Cuda:

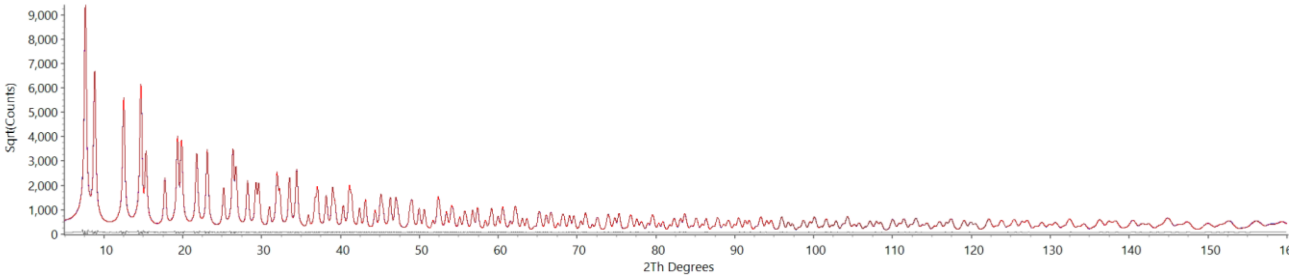

9.2 Crystallite size, microstrain, and thermal displacements

Cuda:

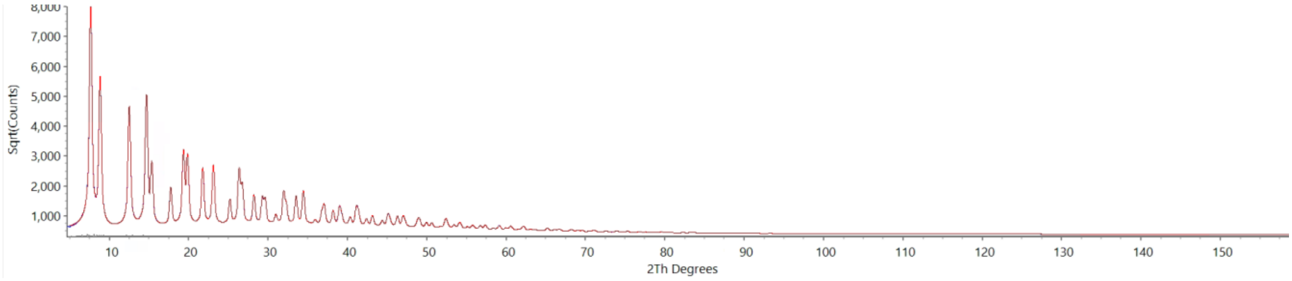

Supplement: Supplementary file 1 [file a-81-00401-sup1.pdf]
